# Controlled Irrigation and Drainage Reduce Rainfall Runoff and Nitrogen Loss in Paddy Fields

**DOI:** 10.3390/ijerph18073348

**Published:** 2021-03-24

**Authors:** Yanmei Yu, Junzeng Xu, Pingcang Zhang, Yan Meng, Yujiang Xiong

**Affiliations:** 1 College of Water Conservancy and Hydropower Engineering, Hohai University, Nanjing 210098, China; yanmeiyuhljsky@sina.com (Y.Y.); zhangpc@mail.crsri.cn (P.Z.); 2 College of Agricultural Science and Engineering, Hohai University, Nanjing 210098, China; hljskymy@163.com; 3 Changjiang River Scientific Research Institute, Wuhan 430010, China; yujiangxiong@126.com

**Keywords:** controlled irrigation and drainage, rainfall runoff, nitrogen loss, paddy field

## Abstract

In southern China, the growing period of rice is synchronized with the rainy period, and the loss of nutrients (such as nitrogen) due to unreasonable irrigation and drainage, along with rainfall and runoff, has become the main source of agricultural nonpoint source pollution. The laws of runoff and nitrogen loss in paddy fields under different irrigation and drainage modes are not clear. In this study, field experiments were adopted to observe the runoff and nitrogen loss under typical rainfall and throughout the whole growth period. The results showed that, compared with the traditional irrigation and drainage mode, the controlled irrigation and drainage mode reduced the drainage of two typical rainfall processes by 47.5% and 31.3% and the peak drainage by 38.9% and 14.4%. Compared with those under the traditional irrigation and drainage mode, the average concentrations of total nitrogen, nitrate nitrogen, and ammonium nitrogen under the controlled irrigation and drainage mode were reduced by 22.2%, 22.7%, and 27.8%, respectively, during the whole rainfall process on July 21 and were decreased by 27.1%, 11.4%, and 25.6%, respectively, on August 25. In irrigated rice areas, under the controlled irrigation and drainage mode, drainage was reduced after two intercepts through paddy fields and drainage ditches. The nitrogen concentration in the drainage ditch decreased due to the increase in retention time and the effect of the ditch and field wetland. Compared with the traditional irrigation and drainage mode, the total nitrogen, nitrate nitrogen, and ammonium nitrogen loads of the controlled irrigation and drainage mode were reduced by 69.8%, 65.3%, and 69.7%, respectively.

## 1. Introduction

Rice is the most important grain crop in China, accounting for 29% of the world’s output and 19% of the world’s planted area [1]. In southern China, where water resources are abundant, the uneven distribution of rainfall throughout the year and the rapid growth of domestic water use in industry, towns, and villages have aggravated the water shortage [2,3,4]. Under the increasingly acute contradiction of water resource supply and demand, implementing water-saving irrigation, and using existing water resources efficiently to promote the virtuous circle of society and economy are important tasks, and inevitable choices for the rational development and utilization of agricultural water resources. However, in recent years, the loss of nitrogen and other nutrients caused by excessive fertilization in paddy fields to maintain rice yield and stable development has become the main source of agricultural nonpoint source pollution in southern China [3,4,5,6,7]. Under traditional irrigation and drainage modes, the high-concentration nitrogen pollutants in the drainage of paddy fields are directly discharged without any treatment because most paddy fields are open and freely drained. Especially in the early stage of the rainy season, the increase in suspended matter in surface water causes the loss of nitrogen in runoff due to the hydraulic erosion effect of raindrops on the surface of paddy fields [8,9,10,11,12]. This loss not only reduces soil fertility and the effective utilization rate of fertilizer, but also causes eutrophication and other water pollution problems.

In paddy fields, nitrogen is mainly transported by water flow between the soil and water medium, so various water management measures have an impact on nitrogen loss. With the development of agricultural irrigation technology, an increasing number of water-saving irrigation methods have been applied to the water management of paddy fields, such as controlled irrigation (CI), furrow irrigation (FI), and alternate wetting and drying (AWD) [13]. Compared with traditional irrigation (TI), these water-saving irrigation methods can not only save 20–30% of irrigation water but also greatly reduce runoff and nitrogen losses in paddy fields [2,6,13,14]. By controlling soil moisture, CI not only reduces the amount of irrigation water but also promotes the absorption of water and fertilizer nutrients by rice plants, and the nitrogen concentration discharged from the field is lower than that under TI [15]. Peng et al. [2] showed that the concentrations of total nitrogen (TN), ammonium nitrogen (NH_4_^+^-N), and nitrate nitrogen (NO_3_^−^-N) in drainage during the whole growth period were 12.1%, 20.3%, and 13.5% lower under CI than under TI, respectively. Xiong et al. [16] found that compared with TI, CI reduced water displacement by 54.2%, TN concentration by 11.5%, and TN load by 54%. By comparing the differences between CI and TI, Yang et al. [17] found that CI could promote nitrogen migration from surface water to the soil, reducing the TN concentration in paddy fields. However, under water-saving irrigation, the oxygen content and nitrification in the soil increased due to the constant changes in the dry and wet conditions of the paddy soil, which aggravated nitrate nitrogen loss in the paddy fields. Compared with TI, CI increased nitrate nitrogen loss by 8.99–16.0%. Cui et al. [18] showed that water-saving irrigation might increase NH_4_^+^-N and NO_3_^−^-N concentrations in percolation water and reduce total percolation water compared with TI. Nitrogen loss from paddy fields was also lower under water-saving irrigation than under TI. Qiao et al. [11] found that the concentration of TN in drainage was 14.8% higher under water-saving irrigation than under TI, but the drainage of water-saving irrigated rice fields was lower. Therefore, water-saving irrigation effectively controlled the total emission of TN in paddy fields.

Controlled drainage (CD) is also recognized as the best management practice to reduce the transport and delivery of nitrogen to sensitive surface water [19,20,21]. There are two ways to reduce nitrogen loss in paddy fields under drainage conditions. One is to reduce drainage, and the other is to reduce the nitrogen concentration in the drainage. Using ten years of data collected from an agricultural drained field in eastern Indiana with two sets of paired plots, Samaneh et al. [22] found that CD plots had statistically significantly (at the 5% level) lower annual drain flow (eastern pair: 39%; western pair: 25%) and nitrate load (eastern pair: 43%; western pair: 26%) compared with free draining (FD) plots. Other studies have reported similar results. Ross et al. [23] found that CD reduced annual drain flow by 46% and annual nitrate loads by 48%. Peng et al. [6] and Yang et al. [3] found that CD could effectively reduce the amount of water and the loss of pollutants. The significant decrease in the NO_3_^−^-N load has been attributed to reduced drainage flow rather than changes in the NO_3_^−^-N concentration [10,24]. However, Ng et al. [25] found that the concentration of nitrogen in drainage was reduced by more than 36% in sandy loam, even though the drainage volume was not reduced by the subsurface pipe.

In contrast to the extensive results available for water-saving irrigation or controlled drainage in paddy fields, information on runoff and nitrogen losses under controlled irrigation and drainage is scarce. In particular, the features of water and nitrogen loss in rice fields during rainfall runoff have not been reported. When drainage is controlled jointly by paddy fields and ditches, the rainfall is intercepted first by the paddy field and then by the ditch. The wetland effect of paddy fields and drainage ditches should reduce nitrogen concentrations and may have a better emission reduction effect than paddy fields or drainage ditches alone. In this paper, field experiments were performed to analyze the drainage process and the rule of nitrogen loss in paddy fields under individual rainfall events, and the contribution of rainfall to nitrogen loss from paddy fields was studied to provide a scientific basis for protecting the ecological environment of farmland and preventing nonpoint source pollution in irrigation areas. Our goal was to verify the feasibility of controlled irrigation and drainage technology by combining drainage ditches and fields. This paper analyzes the rules of water and nitrogen loss during the whole growth period of rice under different irrigation and drainage modes and is expected to provide a basis for water-saving and emission-reduction theory and technology development in rice-growing areas of southern China.

## 2. Materials and Methods

### 2.1. Experimental Site

The study was conducted in paddy fields in Zhoubeidun Village in the Gaoyou Irrigation District of China (32°45′55″ N, 119°11′15″ E) in 2018. The region has a subtropical monsoon climate, with an average annual air temperature of 14.6 °C, an average annual sunshine of 2208 h, and a frost-free period of 222 days per year. The mean annual precipitation is 1037 mm. The soil in the experimental site is dark-yellow hydromorphic paddy soil, which is heavy loam in the plowed layer. The saturated soil water content (vol·vol^−1^) in the 0–20, 0–30, and 0–40 cm layers was 55.1%, 52.7%, and 51.8%, respectively. The organic matter, total nitrogen, total phosphorus and total potassium were 30.3, 1.8, 1.4, and 20.9 g·kg^−1^, respectively, and the pH (soil/H_2_O = 1:2.5) was 7.4.

### 2.2. Experimental Design

**Figure 1:**
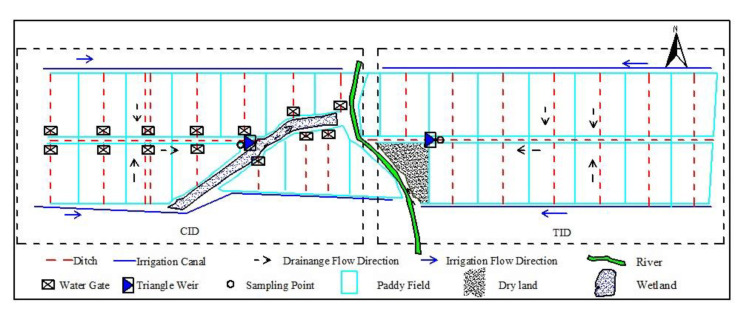
The layout of the study area.

The experiment comprised two irrigation treatments, namely, traditional flooding irrigation (TI) and controlled irrigation (CI), and two drainage treatments, namely, traditional drainage (TD) and controlled drainage (CD). The two treatment combinations were the traditional irrigation and drainage (TID, TI+TD) and the controlled irrigation and drainage (CID, CI+CD). TID was conducted on 7.20ha located on the east side of the river. CID was conducted on 7.90ha located on the west side of the river (Figure 1).

Traditional irrigation (TI) is a common practice and here refers to the supply of irrigation water in rotation for 15 h every four days during the regreening stage and the early tilling stage, 15 h every five days during the late tilling, jointing–booting, and heading–flowering stages, and 20 h every six days during the milking and ripening stages if there is no rainfall before a scheduled irrigation event. After transplanting seedlings, the controlled irrigation management retained a thin water layer of 5–25 mm during the regreening stage and no water layer during the remaining growth stages. The irrigation time and irrigation quota were determined by taking soil moisture in the root layer as a control index (Table 1).

TD was carried out according to local customs, and an open ditch was used for free drainage. In CD, gates were placed in drainage ditches in the paddy fields and were designed to overflow automatically when the water was fully stored. Except for the late tillering stage and the yellow maturity stage, water directly drained to the 10cm water level (ecological water level) of the drainage ditch. The ecological water level was maintained to ensure the normal growth of plants in the drainage ditch. In the other stages, the lowest elevation of the field was taken as the upper limit of drainage. The ecological water level of the drainage ditch met the requirements of the lowest ecological water level of the drainage ditch.

The rice variety was Zhen 99, a type of Japonica rice that is predominantly cultivated in the Gaoyou region. The rice seedlings were transplanted to paddy fields on June 14 and harvested on October 13. The average total growing period was 135 days. The paddy fields of both CID and TID received the same fertilization management (Table 2).In the experiment, urea(nitrogen content ≥46.2%) was applied in four times, namely basal fertilizer before rice transplanting, topdressing in the early tillering stage (June20), the late tillering stage (July20) and the jointing stage (August5), and the amount of fertilizer applied was calculated as pure nitrogen, with a total of 480 kg N ha^−1^. In addition, 25 kg P_2_O_5_ ha^−1^ phosphate fertilizer and 10 kg K_2_O ha^−1^ potassium fertilizer were applied.

### 2.3. Field Measurements

The stage-discharge relation was used to monitor the irrigation amount. The flow velocities at different metering points to determine the parameters of the stage-discharge relation measured using velocity area method and a propeller flow meter. Water level at the inlet of the canal was recorded using a water level recorder, and the seasonal irrigation amount for each canal was calculated by stage-discharge relation and the monitored water levels. Daily water depth and soil moisture of typical fields were measured by vertical ruler and frequency domain reflectometer to schedule irrigation.

A small automatic meteorological observation station (Watchdog2000) and rain gauge cylinder (Hobo) were used for rainfall observations, and the data collection frequency was 1 h. A triangular weir (90°) was installed at the outlet of the drain. The change in the outlet water of the drain pipe was recorded using a recording water level gauge (Odyssey, New Zealand). The Weir flow formula was used to calculate the discharge of the drain as follows:

(1) $$Q=1.343\times H^{2.47}$$

where *Q* is the flow, cm^3^ s^−1^, and *H* is the head of water, m.

### 2.4. Water Sampling and Analysis

At the rainfall events (21 July and 25 August), drainage water samples were collected manually in 500mL plastic bottles, at the outlet of the triangular weir of the drainage ditch. They were collected per hour after rainfall began. To investigate the changes in nitrogen concentrations of drainage water during the whole growth stage, drainage water samples were collected from 14 June to 13 October in 500mLplastic bottles at the outlet of the triangular weir of the drainage ditch. Each water sample had three replicates. After collection, all samples were stored in a container with ice and transported to the laboratory.

The total nitrogen (TN), ammonia nitrogen (NH_4_^+^-N) and nitrate nitrogen (NO_3_^−^-N) concentrations in the water samples were analyzed by the alkaline potassium persulfate digestion method, indophenol blue method, and disulfonic acid-phenol method, respectively (MEPC 2002). A UV spectrophotometer (Cary 50 Spectrophotometer, Varian, Palo Alto, CA, USA) was used for measurement. The average value of nitrogen in water sample was calculated by three replicates.

### 2.5. Statistical Analyses

The Microsoft Excel 2016 was used to process the test data. Regression analysis was performed to determine the relationships between rainfall or irrigation and drainage or nitrogen load. Regression analysis and other statistical analysis were performed using SPSS 17.0 software (IBM SPSS Statistics, Chicago, IL, USA).

## 3. Results

### 3.1. Runoff during Individual Rainfall

**Figure 2:**
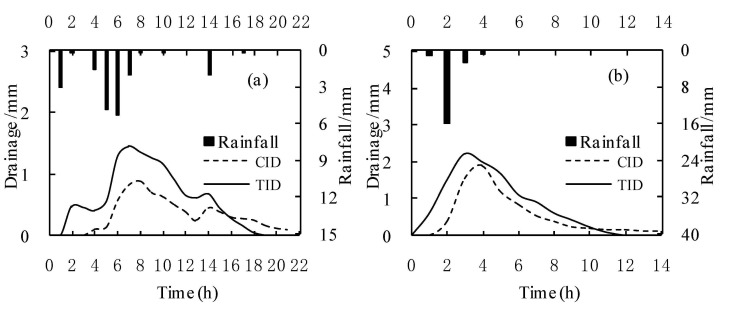
Individual rainfall runoff on 21 July (**a**) and 25 August (**b**).

Two typical rainfall events on 21 July (Figure 2a) and 25 August (Figure 2b) were analyzed to assess the influence of different irrigation and drainage modes on the drainage of paddy fields during the rainfall process. The drainage trends of paddy fields with different irrigation and drainage modes were basically the same after rainfall. As rainfall occurred, the drainage started to rise gradually and reached a peak value; after rainfall, it gradually decreased. The total rainfall was 19.8 mm and 20.1 mm in the two typical rainfall processes, respectively. Under the CID mode, runoff in the drainage ditches started at 4 h and 2 h after rainfall in the two typical rainfall processes, and the drainage was 7.1 mm and 7.8 mm, respectively, 47.5% and 31.3% lower than the TID mode. Compared with the TID mode, the drainage peak values of the CID mode were reduced by 38.9% and 14.4%, and the effect of peak weakening was significant. This effect occurred because the paddy fields under the CID mode maintained a long waterless state during the rice growth period, which increased the effective rainfall and reduced the surface runoff of the paddy fields after rainfall [26]. Hitmi et al. [27] found that the drainage of water-saving irrigation in paddy fields was 27% of the traditional irrigation because water-saving irrigation increased the rain storage capacity and reduced the rainfall runoff. In addition, the soil of the paddy fields under the CID mode was in the unsaturated state for a long time, and the time of alternating wet and dry states between paddy fields and drainage ditches was reduced, which greatly reduced the vertical and lateral leakage and drainage of the paddy fields [6]. In addition, under the CID mode, a water layer was not retained on the surface of the paddy fields, which reduced the drainage of the paddy fields after irrigation [2,7]. After using the CID mode in the drainage ditch, the residence time of the drainage of the paddy fields in the drainage ditch was prolonged by controlling the drainage process, and the amount of water supplied to the paddy fields through lateral seepage increased [28,29]. In addition, the CID mode raised the water level of the outlet of the drainage ditch and reduced the hydraulic gradient of runoff and the drainage velocity of the drainage ditch, which made the drainage process gentler and reduced the erosion of the drainage ditch [20,22,30]. Under CID, the drainage of paddy fields decreased significantly, and the effect of emission reduction was obvious.

### 3.2. Nitrogen Loss during Individual Rainfall

**Figure 3:**
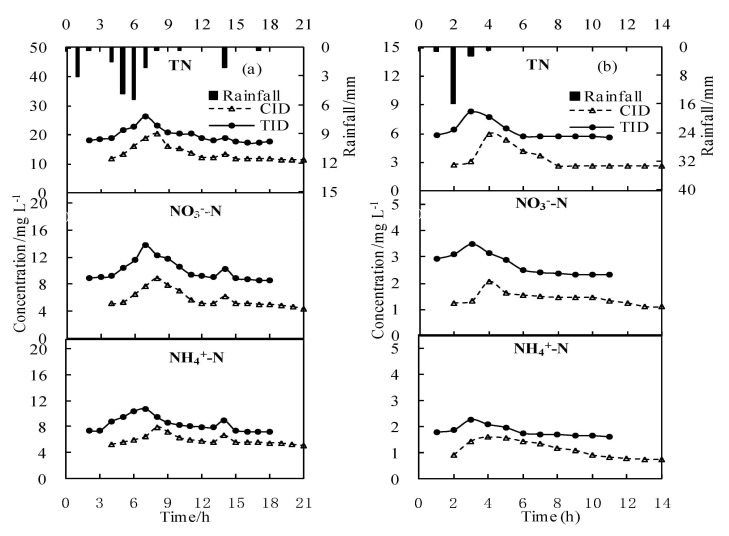
Nitrogen loss during the individual rainfall events on 21 July (**a**) and 25 August (**b**).

The changes in TN, NO_3_^−^-N, and NH_4_^+^-N concentrations in water under the different irrigation and drainage modes were basically consistent (Figure 3). The effects of rainfall on the leaching of nitrogen from soil and the dilution of nitrogen from runoff occurred simultaneously. At the beginning of rainfall, the dissolved leaching effect of rainfall on soil nitrogen was dominant, and the nitrogen concentration carried by runoff increased until it reached a peak. Thereafter, the nitrogen concentration gradually fell due to the dilution effect of runoff. The nitrogen concentration was lowest at the end of the rainfall and tended to be stable thereafter. Generally, nitrogen loss increased due to rainfall erosion in paddy fields, which led to increased nitrogen output in drainage ditches. It takes time for rainfall to interact with the soil to remove nitrogen. As the rainfall proceeded, the utilization of nutrients in paddy fields increased, the nitrogen that could be carried away by exchange decreased, and the output of nitrogen to be lost to the ditch system gradually leveled off [31], as shown in the test results.

Compared to the TID mode, the average concentrations of TN, NO_3_^−^-N, and NH_4_^+^-N under the CID mode on 21 July were reduced by 22.2%, 22.7%, and 27.8%, respectively. In the whole rainfall process on 25 August, the values decreased by 27.1%, 11.4%, and 25.6%, respectively. The TID mode delayed the drainage time of paddy fields to drainage ditches during rainfall by controlling the drainage outlet. Previous studies showed that the TID mode extended the residence time of rainwater in paddy fields and that the drainage time lagged that in the CID mode by approximately six hours [31]. The CID mode increased the residence time of rainwater in paddy fields and settled some soil particles in the rainwater to reduce the loss of nitrogen. The extension of drainage also increased the infiltration of rainwater in paddy fields and reduced the nitrogen concentration in the drainage due to filtration and adsorption by the soil [4,5,7,32]. The CID mode extended the residence time of water in the ditch by controlling and adjusting the drainage process, and the drainage time was one hour longer than that of the TID mode. The initial concentrations of TN, NO_3_^−^-N, and NH_4_^+^-N in the drainage under the CID mode decreased by 24.2%, 19.7%, and 18.8% compared with the TID mode on 21 July, and by 14.9%, 25.6%, and 30.8% on 25 August. Studies have shown that the nitrogen concentration can be reduced by more than 50% when drainage is delayed for two hours in the drainage ditch after runoff production [4]. Zhang et al. [33] showed that the TN concentration decreased by 18.3%, 38.9%, 84.9%, and 85.6% when the residence time of source sewage in the constructed wetland system was 0.5, 1, 2, and 3 days, respectively. Therefore, the CID mode reduces nitrogen emissions by increasing the residence time of rainwater in paddy fields and drainage ditches, which is beneficial to give full play to the wetland function of drainage ditches through soil particle precipitation, soil adsorption, plant absorption, and denitrification.

The peak value of nitrogen concentration occurred at the initial stage of rainfall. Wang et al. [12] and Obermann et al. [34] found that the surface nitrogen of paddy fields was quickly transported to runoff due to the scouring effect of the initial rainfall. The maximum values of the TN, NO_3_^−^-N, and NH_4_^+^-N concentrations occurred at 7 h (21 July) and 3 h (25 August). In the CID mode, these values occurred 1 h later than in the TID mode (Figure 3). As the rainfall continued, the erosion effect of raindrop splashing decreased gradually, and the nitrogen concentration in the surface drainage decreased rapidly. Therefore, controlling surface drainage in the initial stage of rainfall is an important way to reduce nitrogen emissions in paddy fields. During the whole rainfall process, the peak concentrations of TN, NO_3_^−^-N, and NH_4_^+^-N were 35.9%, 48.3%, and 24.8% lower in the CID mode than in the TID mode on 21 July and 27.7%, 20.7%, and 29.8% lower on 25 August, respectively. By controlled irrigation and drainage technology to retain the initial rainfall and increase the residence timeof rainwater, the drainage and nitrogen concentration of paddy fields can be effectively reduced.

### 3.3. Runoff Traits

**Figure 4:**
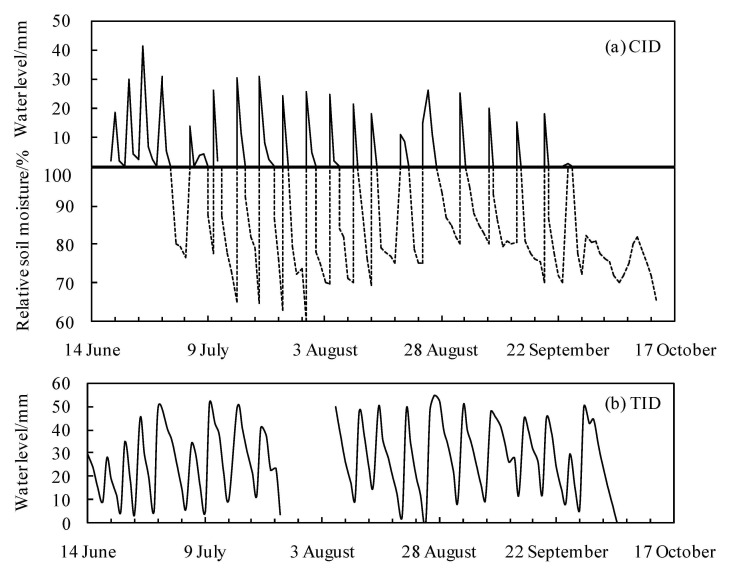
Typical water depth and soil moisture conditions in the controlled irrigation and drainage (CID) mode (**a**) and the traditional irrigation and drainage (TID) mode (**b**).

Sixteen wet–dry cycles occurred in the CID paddy fields, with 94 days of non-flooding conditions (Figure 4a). For the TID mode, ponding was maintained except from 26 July to 2 August. This period corresponded to drainage in the late tillering stage to restrain nonproductive tillering (Figure 4b).

**Figure 5:**
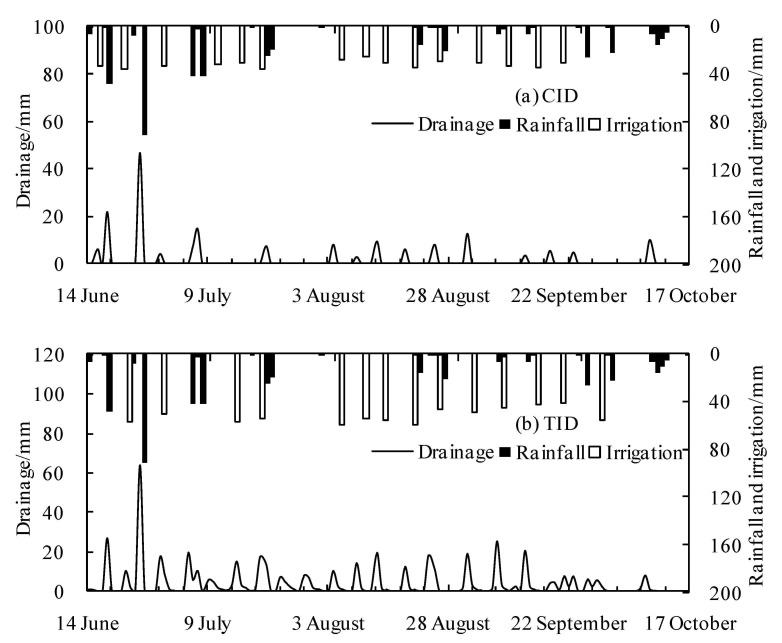
Typical daily rainfall, irrigation and drainage. (**a**) Controlled irrigation and drainage (CID) mode and (**b**) the traditional irrigation and drainage (TID) mode.

The irrigation amounts in the CID and TID modes were 481.2 mm and 726.3 mm, respectively (Figure 5). There were 24 rainfalls during the rice growth period in the test area, and the total rainfall was 428.2 mm, including four rainstorms (≥30 mm) and seven heavy rain events (15–29.9 mm). In the CID and TID modes, the numbers of drainage were 16 and 21, respectively, and the drainage were 175.5 mm and 446.9 mm, respectively. Effective controlled drainage of paddy fields is an important way to reduce agricultural nonpoint source pollution [19,35,36]. Compared with the TID mode, the CID mode significantly reduced drainage by 271.4 mm and60.7%. In the CID mode, drainage was primarily concentrated in the regreening period (from 14 June to 25 June), and less drainage occurred in the rest of the growth period. Under the TID mode, water drainage was maintained at a high level in each growth stage, and the distribution was more uniform. During plum rain in the early stage of rice growth, the coverage of rice in the field was small, and rainstorms were frequent. In addition, the more nitrogen fertilizer there is, the higher the risk of nitrogen loss in the early stage of rice growth under the action of raindrop splash erosion and runoff erosion [2,7,37,38]. During this period, the drainage of the CID mode decreased by 29.5 mm and 28.4% compared with the TID mode. The CID mode reduced the drainage of nitrogen in the critical period and effectively controlled nonpoint source pollution by paddy field drainage to the surrounding water.

### 3.4. TN, NO_3_^−^-N, and NH_4_^+^-N Load by Runoff

**Figure 6:**
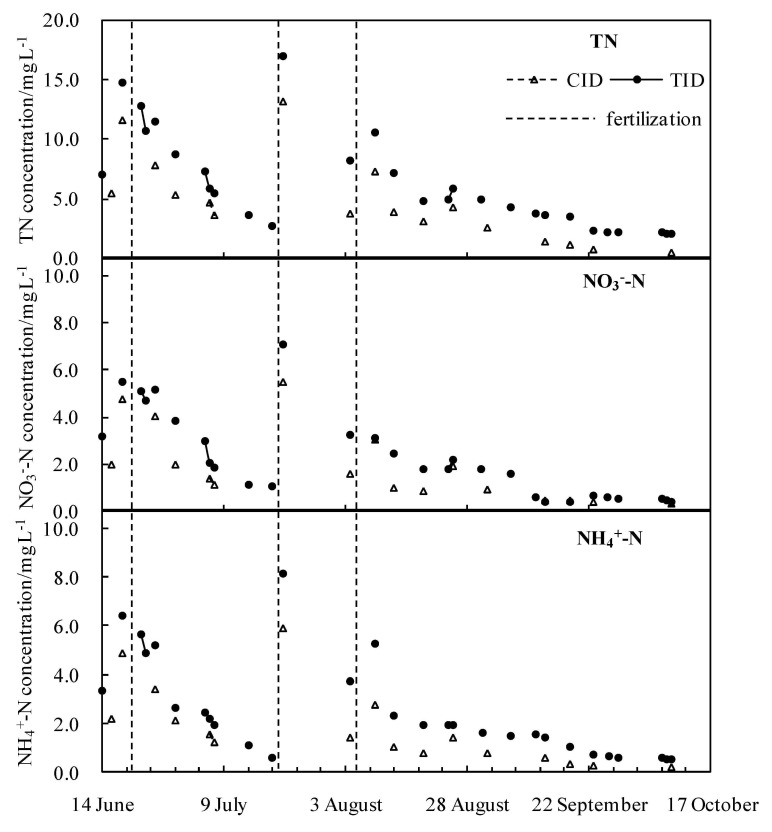
Nutrient concentration of drainage water sampled at the outlets of the CID and TID modes.

The nitrogen concentration peaked many times during the whole growth period (Figure 6). The concentrations of TN, NO_3_^−^-N, and NH_4_^+^-N in the drainage changed uniformly; they were higher in the early growth stage of rice, gradually decreased after entering the jointing and booting stage (5 August) and remained stable at the end of the milking stage. In the total growth stage, the average concentrations of TN, NO_3_^−^-N, and NH_4_^+^-N in drainage treated by the CID mode were 25.5%, 19.1%, and 28.3% lower than those treated by the TID mode, respectively. Compared with the TID mode, the CID mode controlled the water level in the ditch at a higher level by raising the water level at the outlet of the drainage ditch and extending the drainage time of the ditch. A large part of the suspended load and bed load in the drainage were deposited in drainage ditches, allowing the nitrogen in the drainage to be fully absorbed by sediment and aquatic plants. As a result, the nitrogen concentration in the drainage was greatly reduced [3,25,39].

In the CID mode, paddy field drainage was reduced after two intercepts of paddy fields and drainage ditches (Table 3). The increased residence time enhanced the wetland effect of the paddy fields and reduced the nitrogen concentration in the drainage ditch. The nitrogen load was lower in the CID mode than in the TID mode at each growth stage, particularly in the early growth stage (the regreening, the tillering, the jointing and booting).As a result, the TN, NO_3_^−^-N, and NH_4_^+^-N loads were lower than those in the TID mode by 69.8%, 65.3%, and 69.7%, respectively. Under the CID mode, the loads peak of the TN, NO_3_^−^-N, and NH_4_^+^-N occurred in the regreening stage decreased by 51.1%, 44.1%, and 53.5%, respectively. Due to the relatively high temperature and low rice coverage when the basal fertilizer was applied, the ability of rice roots to absorb nitrogen fertilizer was relatively poor. At this time, the rainfall is relatively frequent, the most of the nitrogen was discharged with the surface runoff before it can absorbed by the root system, so the nitrogen load in the drainage was high. At this stage, reducing drainage or avoiding drainage as much as possible is the key to reducing nitrogen loss in farmland. Extensive variations in the effects of the CID mode on drainage and nitrogen load at different sites have been reported in the literature. Zhu et al. [7] showed that the displacement and total nitrogen load of the CID mode were 60.6% and 58.6% lower than that of the TID mode at the farmland scale. At the drainage ditches scale, the CID mode reduced drainage and total nitrogen by 55.9% and 59.7%, respectively, compared with the TID mode. Peng et al. [6] showed that the CID mode had significant environmental effects by reducing NO_3_^−^-N (59.2%) and NH_4_^+^-N (45.2%) leaching losses from paddy fields through reduced water leakage. Although the paddy fields and drainage ditches had different water residence time rates under different meteorological, soil type, tillage system, and drainage design conditions, water displacement and the nitrogen concentration in drainage were obviously reduced. Therefore, compared with the TID mode, the CID mode effectively reduces pollution of downstream water bodies by reducing nitrogen emissions from paddy fields and improving the utilization rate of farmland nutrients and rainwater resources.

## 4. Discussion

### 4.1. Nitrogen Loss and Its Relationship to Rainfall

**Figure 7:**
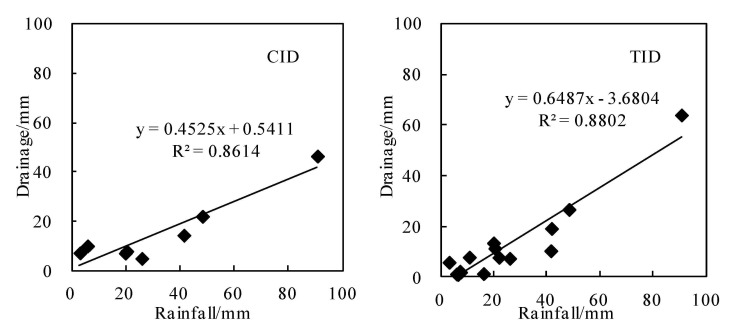
Regression analysis of rainfall and runoff using daily data.

Surface runoff caused by rainfall is one of the main routes of water loss in paddy fields [7]. Jung et al. [40] showed that the daily runoff is mainly controlled by rainfall rather than irrigation. A positive correlation between rainfall and runoff during runoff events was observed in 2004 (R^2^ = 0.90) and 2005 (R^2^ = 0.83). Cho and Han [41] observed a significant correlation (R^2^ > 0.65) between rainfall and runoff in Korean paddy fields. In this study, there was a positive correlation between rainfall and drainage during the whole growth period by the regression analysis of rainfall and drainage under different irrigation and drainage modes (R^2^ = 0.8614 and R^2^ = 0.8802) (Figure 7). The correlation between irrigation and drainage (R^2^ = 0.032 and R^2^ = 0.3195) was poor, which indicated that the drainage of paddy fields was mainly discharged as rainfall runoff. These research results are basically consistent with those described in the above literatures. China’s rice planting area exceeds 30 million ha, which is mainly distributed in the southern region, where the growth period is synchronized with the rainy period. Compared with the TID mode, the CID mode reduced surface runoff caused by rainfall by 34.3%. Therefore, the CID mode can effectively reduce runoff, which is of practical significance for reducing downstream drainage and flood control pressure.

**Figure 8:**
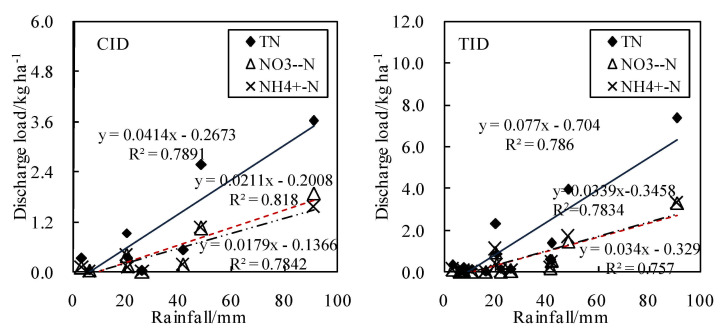
Regression analysis of rainfall and discharge load using daily data.

Surface runoff formed by rainfall was an important way for nitrogen loss. Grazhdani et al. [10] found that most nitrogen (50%) was lost during heavy rainfall. Studies have shown that rainfall runoff within a short period of time carries a large number of nutrients into surrounding water bodies, especially after fertilizer application, resulting in serious water eutrophication [9,12,40]. Arheimer and Liden [8] found that the concentration of inorganic nitrogen in drainage was increased, and the increased nitrogen concentration related to the multiple precipitation events. Qiao et al. [11] found that nitrogen loss caused by runoff was linearly positively correlated with nitrogen application and was seriously affected by rainfall. Rainfall during the whole growth period was positively correlated with the TN by the regression analysis of rainfall and nitrogen load under different irrigation and drainage modes, TN (R^2^ = 0.7891 and R^2^ = 0.7867), NO_3_^−^-N (R^2^ = 0.818 and R^2^ = 0.7834), and NH_4_^+^-N (R^2^ = 0.7842 and R^2^ = 0.7574) (Figure 8). The correlation between irrigation and N loss was poor for TN (R^2^ = 0.0402 and R^2^ = 0.0003), NO_3_^−^-N (R^2^ = 0.147 and R^2^ = 0.032), and NH_4_^+^-N (R^2^ = 0.0542 and R^2^ = 0.021), which indicated that runoff loss caused by rainfall was the main form of nitrogen loss in paddy fields. This was basically consistent with the conclusions of the above-mentioned scholars. The number of nitrogen loss caused by rainfall in the CID mode decreased by seven times compared with the TID mode during the whole growth period (Table 4). The TN, NO_3_^−^-N, and NH_4_^+^-N decreased by 52.6%, 47.7%, and 54.1%, respectively, and the peak value decreased by 50.9%, 43.2%, and 52.7%. The CID mode significantly reduces the nitrogen loss load caused by rainfall.

### 4.2. Optimizing Water Management

Three fertilization events (20 June, 20 July, and 5 August) resulted in significantly increased mass concentrations of TN, NH_4_^+^-N, and NO_3_^−^-N in the drainage (Figure 6). On 21 July, the nitrogen concentration in the rainfall runoff on the second day after fertilization increased significantly, but it did not change much on 25 August, which was 20 days from the nearest fertilization interval. In addition to fertilizer application, the time interval between rainfall and fertilization is also an important factor affecting nitrogen concentration in rainfall runoff. Other scholars have reached similar conclusions. Qiao et al. [11] monitored the nitrogen loss in paddy field runoff at a fixed point. Under the same fertilizer treatments, the nitrogen loss in 2009 (57 kg ha^−1^) was greater than that in 2008 (15 kg ha^−1^). The main reason for this difference was that the rainfall time of runoff generation in 2008 was mostly far from the fertilization time; thus, at the time of rainfall, the nitrogen concentration in the field water had dropped to a low level. The shorter the interval between rainfall and fertilization, the greater the nitrogen loss carried away by the runoff process and the effect of fertilization on the nitrogen concentration. Therefore, it was necessary to avoid fertilizing before the arrival of heavy rainfall and to adjust the drainage time at the initial stage of rainfall reasonably, which was helpful to slow down agricultural nonpoint source pollution emissions. When the interval between rainfall and fertilization was more than 1 week, fertilization had little effect on the nitrogen concentration. Therefore, the first week after fertilization is an important pollution control period for rice fields, and drainage of rice fields should be minimized [42]. Xiao et al. [43] showed the optimal time for surface drainage by established a multi-objective controlled drainage model were the fourth, seventh, seventh, and fifth day at the stage of the tillering, the jointing and booting, the earing and sprouting, and the milking after flooding, respectively. A field survey found that the impact of water management on nitrogen loss was greater than that of fertilization, especially after fertilization [27,42,44]. After fertilization, the CID mode effectively reduced the nitrogen concentration in the drainage of the paddy fields by reducing the downstream nitrogen discharge from the drainage ditches, which effectively avoided pollution of downstream water bodies.

## 5. Conclusions

The loss of water and nitrogen in paddy fields is mainly caused by rainfall runoff, and runoff in a short time after fertilizer application carries a large number of nutrients into the surrounding water bodies, resulting in serious nonpoint source pollution. The analysis of two typical rainfall runoff and nitrogen concentration variation rules revealed that the CID mode stored the initial rainfall in paddy fields and drainage ditches, which reduced the peak water flow after rainfall and increased the retention time of rainwater. The peak reduction effect was significant, which was beneficial for the wetland effect of drainage ditches and rice fields. Compared with the TID mode, the CID mode reduced the drainage caused by rainfall, especially the drainage of paddy fields in the early stage of rice growth, which effectively reduced the drainage and the nitrogen concentration carried from paddy fields into the downstream water. The CID mode can effectively reduce nitrogen emissions from paddy fields and improve the utilization rate of farmland nutrients and rainwater resources, thereby reducing the pollution of downstream water bodies.

## Figures and Tables

**Table 1:** Soil moisture thresholds for controlled irrigation in the different stages.

Limit	G ^a^	T			J/B		E/S	M	Y
		Early	Middle	Late	Early	Late			
Upper	25 mm ^b^	θs_1_	θs_1_	θs_1_	θs_2_	θs_2_	θs_3_	θs_3_	Drying
Lower	5 mm	70%θs_1_	65%θs_1_	60%θs_1_	70%θs_2_	75%θs_2_	80%θs_3_	70%θs_3_	
Root observation depth (cm)	—	0–20	0–20	0–20	0–30	0–20	0–30	0–40	—

^a^ G represents the regreening stage, T represents the tillering stage, J/B represents the jointing and booting stages, E/S represents the earing and sprouting stages, M represents the milk maturity stage, and Y represents the yellow maturity stage. ^b^ Data show the water depth thresholds during the regreening stage. θs_1_, θs_2_, θs_3_ represent the average saturated volumetric soil moisture for the 0–20, 0–30, and 0–40 layers, respectively.

**Table 2:** Time and amount of fertilization.

Date	Nitrogen Fertilizer (kg N ha^−1^)	Phosphate Fertilizer (kg P_2_O_5_ ha^−1^)	Potassium Fertilizer (kg K_2_O ha^−1^)
10 June	200	25	10
20 June	70		
20 July	140		
5 August	70		
Total	480	25	10

**Table 3:** Runoff losses of various N forms from the CID and TID modes.

Treatment	N Forms	Discharge Load (kg ha^−1^)						
		G	T	J/B	E/S	M	Y	Total
**CID**	**TN**	6.1	1.5	1.2	0.2	0.0	0.1	9.1
	**NO_3_^−^-N**	3.0	0.5	0.4	0.0	0.0	0.0	3.9
	**NH_4_^+^-N**	2.6	0.6	0.4	0.0	0.0	0.0	3.6
**TID**	**TN**	12.6	7.3	6.2	2.2	1.5	0.3	30.1
	**NO_3_^−^-N**	5.3	3.0	2.1	0.8	0.1	0.0	11.4
	**NH_4_^+^-N**	5.6	2.6	2.5	0.7	0.5	0.0	11.9

G represents the regreening stage, T represents the tillering stage, J/B represents the jointing and booting stages, E/S represents the earing and sprouting stages, M represents the milk maturity stage, and Y represents the yellow maturity stage.

**Table 4:** Rainfall runoff losses of various N forms from the CID and TID modes.

Date	Rainfall (mm)	Discharge Load of the CID (kg ha^−1^)			Discharge Load of the TID (kg ha^−1^)		
		TN	NO_3_^−^-N	NH_4_^+^-N	TN	NO_3_^−^-N	NH_4_^+^-N
14 June	6.35				0.08	0.03	0.04
18 June	48.26	2.57	1.05	1.07	3.95	1.47	1.71
23 June	7.37				0.21	0.09	0.10
25 June	90.68	3.63	1.88	1.57	7.38	3.32	3.32
5 July	41.66				1.40	0.57	0.47
6 July	3.05	0.34	0.10	0.11	0.34	0.12	0.13
7 July	41.40	0.53	0.16	0.17	0.57	0.19	0.20
21July	19.81	0.94	0.39	0.42	2.29	0.96	1.10
25 August	20.12	0.34	0.15	0.11	0.90	0.32	0.35
11 September	7.11				0.09	0.01	0.04
23 September	25.88	0.04	0.02	0.01	0.17	0.05	0.05
28 September	21.84				0.17	0.04	0.05
7 October	16.00				0.03	0.01	0.01
8 October	10.67				0.16	0.04	0.04
9 October	5.84	0.05	0.04	0.02	0.03	0.00	0.01
